# Supplementary material for: RNA language models predict mutations that improve RNA function
Source: Nat Commun. 2024 Dec 5;15:10627. doi: 10.1038/s41467-024-54812-y (PMC11621547; doi:10.1038/s41467-024-54812-y)
Supplement: Supplementary file 2 — Description of Additional Supplementary Files [file 41467_2024_54812_MOESM2_ESM.pdf]

Description of Additional Supplementary Files:

Supplementary Data 1: Information about the 231 RNA family dataset within GARNET. The table includes Rfam model IDs, descriptions, and length, as well as the number of Rfam seed and full-alignment sequences. The table also includes the number of GARNET sequences for each Rfam ID, as well as the total GARNET sequence length.

Supplementary Data 2: Optimal Growth Temperatures of GTDB organisms. This dataset contains empirical OGTs from the TEMPURA and Gosha databases, OGTs predicted by TOME, GTDB genome accessions, GTDB taxonomic classifications, and thermal classifications for GTDB isolate descriptions.

Supplementary Data 3: RNA language model parameters and training. All pretrained language models used the following common hyperparameters: batch size = 18, dropout = 0.2, AdamW minimizer beta2 = 0.998, and use of the Flash attention algorithm[63]. Finetuned models had a batch size of 48. Extended parameters are also listed, including specifications for each GNN model.

Supplementary Data 4: Jensen-Shannon divergences of RNA sequences generated by the RNA language models. Nucleotide frequencies of natural sequences in GARNET and generated sequences are included, along with the JSD values at each position. *E. coli* 23S rRNA nucleotide numbering is shown, after removing gaps and insertions (Methods).

Supplementary Data 5: Log likelihood calculations for candidate mutations in *E. coli* 23S rRNA. Values were calculated using the strategy shown in Fig. 6b. Control 3 calculations for all possible single-nucleotide mutations (GNN ΔΔlogP, 23S rRNA LM ΔΔlogP, and 231-RNA LM ΔΔlogP) are included.

Supplementary Data 6: DNA sequences used in this study. Comprehensive list of DNA sequences described in Methods.
