# Supplementary figures and images for: RNA language models predict mutations that improve RNA function
Source: Nat Commun. 2024 Dec 5;15:10627. doi: 10.1038/s41467-024-54812-y (PMC11621547; doi:10.1038/s41467-024-54812-y)

Top gel:

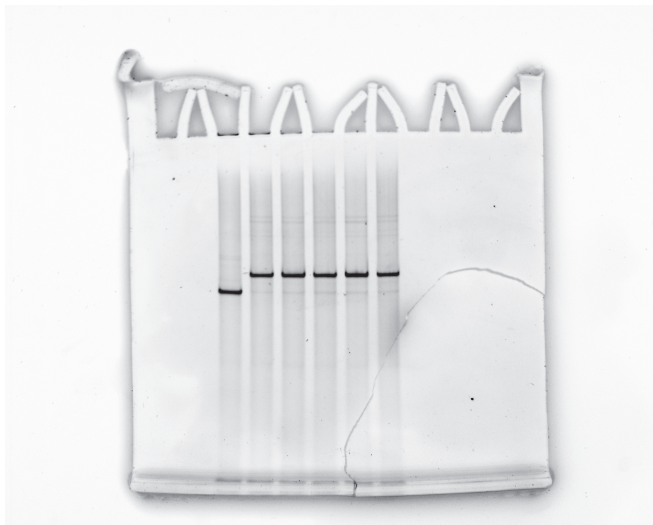

Bottom gel:

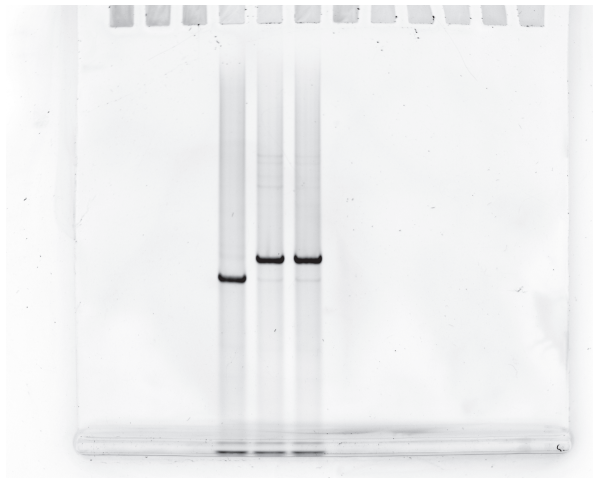

Supplement: Supplementary file 12 — Source Data for Supplementary Fig. 13 [file 41467_2024_54812_MOESM12_ESM.pdf]
